# A machine-learning algorithm for neonatal seizure recognition: a multicentre, randomised, controlled trial

**DOI:** 10.1016/S2352-4642(20)30239-X

**Published:** 2020-10

**Authors:** Andreea M Pavel, Janet M Rennie, Linda S de Vries, Mats Blennow, Adrienne Foran, Divyen K Shah, Ronit M Pressler, Olga Kapellou, Eugene M Dempsey, Sean R Mathieson, Elena Pavlidis, Alexander C van Huffelen, Vicki Livingstone, Mona C Toet, Lauren C Weeke, Mikael Finder, Subhabrata Mitra, Deirdre M Murray, William P Marnane, Geraldine B Boylan

**Affiliations:** a INFANT Research Centre, University College Cork, Cork, Ireland; b Department of Paediatrics and Child Health, University College Cork, Cork, Ireland; c Institute for Women's Health, University College London, London, UK; d Utrecht Brain Center, University Medical Center Utrecht, Utrecht University, Utrecht, Netherlands; e Clinical Neurophysiology, University Medical Center Utrecht, Utrecht University, Utrecht, Netherlands; f Department of Neonatal Medicine, Karolinska University Hospital, Stockholm, Sweden; g Division of Paediatrics, Department CLINTEC, Karolinska Institutet, Stockholm, Sweden; h Rotunda Hospital, Dublin, Ireland; i Royal London Hospital, London, UK; j London School of Medicine and Dentistry, Queen Mary University of London, London, UK; k Department of Clinical Neurophysiology, Great Ormond Street Hospital for Children NHS Trust, London, UK; l Homerton University Hospital NHS Foundation Trust, London, UK

## Abstract

**Background:**

Despite the availability of continuous conventional electroencephalography (cEEG), accurate diagnosis of neonatal seizures is challenging in clinical practice. Algorithms for decision support in the recognition of neonatal seizures could improve detection. We aimed to assess the diagnostic accuracy of an automated seizure detection algorithm called Algorithm for Neonatal Seizure Recognition (ANSeR).

**Methods:**

This multicentre, randomised, two-arm, parallel, controlled trial was done in eight neonatal centres across Ireland, the Netherlands, Sweden, and the UK. Neonates with a corrected gestational age between 36 and 44 weeks with, or at significant risk of, seizures requiring EEG monitoring, received cEEG plus ANSeR linked to the EEG monitor displaying a seizure probability trend in real time (algorithm group) or cEEG monitoring alone (non-algorithm group). The primary outcome was diagnostic accuracy (sensitivity, specificity, and false detection rate) of health-care professionals to identify neonates with electrographic seizures and seizure hours with and without the support of the ANSeR algorithm. Neonates with data on the outcome of interest were included in the analysis. This study is registered with ClinicalTrials.gov, NCT02431780.

**Findings:**

Between Feb 13, 2015, and Feb 7, 2017, 132 neonates were randomly assigned to the algorithm group and 132 to the non-algorithm group. Six neonates were excluded (four from the algorithm group and two from the non-algorithm group). Electrographic seizures were present in 32 (25·0%) of 128 neonates in the algorithm group and 38 (29·2%) of 130 neonates in the non-algorithm group. For recognition of neonates with electrographic seizures, sensitivity was 81·3% (95% CI 66·7–93·3) in the algorithm group and 89·5% (78·4–97·5) in the non-algorithm group; specificity was 84·4% (95% CI 76·9–91·0) in the algorithm group and 89·1% (82·5–94·7) in the non-algorithm group; and the false detection rate was 36·6% (95% CI 22·7–52·1) in the algorithm group and 22·7% (11·6–35·9) in the non-algorithm group. We identified 659 h in which seizures occurred (seizure hours): 268 h in the algorithm versus 391 h in the non-algorithm group. The percentage of seizure hours correctly identified was higher in the algorithm group than in the non-algorithm group (177 [66·0%; 95% CI 53·8–77·3] of 268 h *vs* 177 [45·3%; 34·5–58·3] of 391 h; difference 20·8% [3·6–37·1]). No significant differences were seen in the percentage of neonates with seizures given at least one inappropriate antiseizure medication (37·5% [95% CI 25·0 to 56·3] *vs* 31·6% [21·1 to 47·4]; difference 5·9% [–14·0 to 26·3]).

**Interpretation:**

ANSeR, a machine-learning algorithm, is safe and able to accurately detect neonatal seizures. Although the algorithm did not enhance identification of individual neonates with seizures beyond conventional EEG, recognition of seizure hours was improved with use of ANSeR. The benefit might be greater in less experienced centres, but further study is required.

**Funding:**

Wellcome Trust, Science Foundation Ireland, and Nihon Kohden.

## Introduction

Newborn infants can exhibit a range of unusual repetitive movements, not all of which are seizures.^1^ Recognition of seizures is vital because they are often a sign of an underlying neurological condition such as hypoxic ischaemic encephalopathy, stroke, or meningitis,^2^, ^3^ and because treatment for non-seizure events exposes infants to unnecessary harmful drugs.^4^ The diagnosis of neonatal seizures is challenging for clinicians because most neonatal seizures are electrographic only, clinical signs can become uncoupled after medication, and, even when present, clinical signs can be subtle and hard to distinguish from the normal repertoire of neonatal movements.^5^, ^6^, ^7^, ^8^, ^9^ Amplitude-integrated electroencephalography (aEEG) is often used by neonatologists for seizure detection, but limitations have been reported.^10^ Continuous conventional EEG (cEEG) monitoring is the gold standard for the diagnosis of all seizures.^11^ Evidence suggests that regardless of the underlying cause, seizures themselves have a negative effect on neurodevelopment, adding to the importance of early recognition and treatment.^12^, ^13^, ^14^, ^15^, ^16^ Despite the availability of cEEG in some neonatal intensive care units (NICUs) and the growth of neonatal neurocritical care,^17^ early and accurate diagnosis of seizures and prompt treatment remain a challenge.^18^ Accurate EEG interpretation requires expert input, which is not readily available in most NICUs worldwide, even in high-income countries; it is also expensive and time intensive.^19^, ^20^

**Research in context**

**Evidence before this study**

During development of our machine-learning algorithm (Algorithm for Neonatal Seizure Recognition [ANSeR]) for neonatal seizure detection, we did a systematic review of the scientific literature. We searched PubMed for research articles published in English from inception to Oct 24, 2013, using the following search terms: “automated seizure detection algorithms (SDA) vs gold standard” (221 articles found, of which 27 articles were included), “automated SDA safety” (221 articles found, of which one article was included), and “amplitude-integrated EEG *vs* continuous EEG – interobserver agreement” (87 articles found, of which nine articles were included). We identified six different research groups that assessed the performance of different seizure detection algorithms (SDAs) using at least 100 h of electroencephalography (EEG) monitoring from at least ten neonates. All groups reported performance results on post-acquisition EEG recordings and not in real time in a clinical setting, except for one. This study compared the clinical care of two cohorts of neonates: one cohort using continuous amplitude EEG with SDA output and one cohort of neonates who were clinically monitored with 1 h of conventional EEG monitoring. The SDA detected 55% of seizures but, most importantly, they found that neonates in the SDA cohort did not receive additional doses of antiseizure medication, suggesting that it is safe to use in a real-time clinical setting. The same search was done again on April 25, 2020, to include the 2013–20 period, and we found 297 articles. During this period, only one recent study used a SDA in a neonatal clinical setting. The aim of this study was to assess the feasibility of a monitoring infrastructure proposed for an antiseizure drug trial. The health-care professionals interviewed found the SDA to be useful for seizure detection but with a high rate of false detections. We did not find any clinical trials assessing the impact of a SDA on neonatal seizure recognition by health-care professionals in real time in the neonatal unit.

**Added value of this study**

To the best of our knowledge, the current study is the first randomised, multicentre clinical investigation to assess the clinical impact of a machine-learning algorithm in real time on neonatal seizure recognition in a clinical setting. Although it did not reach our predefined target, using the ANSeR algorithm as a support tool increased the percentage of seizures correctly detected. However, in a post-hoc analysis of the difference between weekdays and weekends, the predefined target was exceeded. This finding might be explained by increased neurophysiology expertise available during the week in the participating neonatal centres. We also demonstrated that the use of ANSeR for seizure recognition was safe and did not result in an increased use of antiseizure medication.

**Implications of all the available evidence**

Our results support the potential benefits and safety for the use of a real-time neonatal seizure detection algorithm. We demonstrated an increase in the percentage of seizures recognised using our algorithm in centres that already had good neurophysiology support or had neonatologists who were comfortable with EEG interpretation. We suggest that the impact might be greater in less experienced neonatal units, although further studies are needed.

One proposed solution has been the incorporation of bedside automated seizure detection software, and several algorithms have been developed for neonates, with varying performance levels reported.^21^, ^22^, ^23^, ^24^, ^25^, ^26^, ^27^ Two of these algorithms—namely, Gotman (Stellate EEG system, Natus Medical, Pleasanton, CA, USA)^21^ and Recognize (Brainz Instruments, Auckland, New Zealand)^25^—have been incorporated into EEG or aEEG systems and are commercially available. A 2019 study used a seizure detection algorithm in the Persyst EEG software (Persyst, Solana Beach, CA, USA) in a clinical setting as part of a proposed monitoring infrastructure for an antiseizure drug trial.^28^ Although health-care professionals found it useful for neonatal seizure detection, they reported a high false detection rate. We have developed an EEG-based seizure detection software system called the Algorithm for Neonatal Seizure Recognition (ANSeR). After repeated training and testing offline, using several datasets of neonatal EEGs,^29^, ^30^, ^31^ we aimed to evaluate the performance of the ANSeR algorithm in real time by assessing the diagnostic accuracy for the detection of neonatal electrographic seizures with and without the use of ANSeR as a support tool for clinicians at the cot side.

## Methods

### Study design and participants

This multicentre, randomised, two-arm, parallel, controlled study was done in eight NICUs across Ireland, the Netherlands, Sweden, and the UK. All neonates between the corrected gestational age of 36 and 44 weeks who were admitted to the NICUs of recruiting hospitals and required EEG monitoring because they had clinically suspected seizures or who were at high risk of seizures were screened for eligibility, and parents or guardians were approached for consent. If written informed consent was not obtained from at least one parent or guardian, the neonate was not included in the investigation.

The trial was a regulated clinical investigation of a medical device. The clinical investigation plan was approved by national competent authorities and local ethics committees of participating centres and adhered to all applicable local and national regulations.

### Randomisation and masking

Eligible neonates were randomly assigned (1:1) to receive cEEG monitoring with the aid of ANSeR (algorithm group) or routine cEEG monitoring alone (non-algorithm group), which is considered to be standard of care in the participating NICUs. Block randomisation (with block sizes of two or four), stratified by recruiting hospital, was used to allocate neonates to each group. The randomisation list was generated by a biostatistician using the ralloc procedure in Stata and incorporated into the central web-based electronic system used for allocation. Because this study was an investigation of a medical device, the research personnel, clinical team, and neonates' families were all aware of group allocation. The neurophysiologists who reviewed the EEGs for post-acquisition seizure annotation and the biostatistician who did the statistical analysis were masked to the group allocation.

### Procedures

For the intervention group, the ANSeR software system ran on a bedside laptop linked to the Nihon Kohden Neurofax monitor (EEG-1200, Tokyo, Japan) and displayed the seizure probability trend in real time. An audible alarm sounded when a predefined probability threshold was breached (0·5), and a red marker was visible on the aEEG display when a possible seizure was detected by the algorithm.^31^ Training for the operation of the ANSeR software system was provided to all personnel involved. Clinical management, including interventions and treatments, of all included neonates was otherwise provided as per standard clinical practice of the recruiting hospitals as the study protocol did not include any instructions regarding seizure treatment.

Neonates in the non-algorithm group were monitored with cEEG using Nihon Kohden Neurofax (EEG-1200), NicoletOne ICU Monitor (Natus, Middleton, WI, USA) or XLTek (Natus). Even though different EEG machines were used for monitoring infants in the non-algorithm group, the EEG montage used was the same for both study groups. EEG recording methodology was standardised across all hospitals using a standard operating procedure, and real-time multichannel cEEG and aEEG traces were displayed at the cot side for clinical review. All hospitals had trained personnel available to start the EEG monitoring and maintain high-quality recordings. Electrodes were positioned according to the 10:20 EEG electrode placement system adapted for neonates, using nine disposable electrodes positioned at F3, F4, C3, C4, Cz, T3, T4, O1, and O2. Separate electrodes were applied for ECG and respiration monitoring and synchronised with the EEG recording. EEG was recorded with a sampling rate of 250 Hz or 256 Hz and a filter bandwidth of 0·5–70 Hz. EEG was performed for a minimum of 2 h and up to 100 h (to include the rewarming period for newborn infants receiving therapeutic hypothermia), or longer if clinically indicated, but the use of the algorithm was only evaluated for the first 100 h of monitoring.

cEEG recordings were reviewed in their entirety, and all EEGs were annotated for seizures twice by independent expert neurophysiologists. One expert (SRM) annotated all EEGs and the second annotation was provided by one of the other experts from the group (GBB, RMP, ACvH, and EP). All expert reviewers adhered to a review protocol specifying reviewing parameters (including montage, sensitivity, and time base) and limiting review periods to prevent fatigue. A neonatal electrographic seizure has been defined as at least 10 s of evolving, sudden, and repetitive stereotyped waveforms on at least one EEG channel.^32^ However, this definition is arbitrary, and we have previously shown that there is poor agreement between experts in seizures with a duration of less than 30 s.^33^ Therefore, to strengthen the experts' annotation as a gold standard, in this investigation an electrographic seizure was confirmed if there was an overlap in annotation of 30 s between two expert reviewers.

Periods during which the two expert annotations overlapped were used to produce a final annotation (ie, the gold standard for seizure detection). For each neonate with seizures, summary measures of seizure burden^14^—total seizure burden in minutes (total accumulated seizure duration in the entire recording), maximum hourly seizure burden (the total seizure burden in the hour with the maximum seizure activity expressed in min/h), and median seizure duration in seconds—were calculated using the final annotations.

For each neonate, the gold standard was the final annotation based on two experts. A neonate was confirmed as having electrographic seizures (neonate with seizure) if there was at least one seizure with an overlap of 30 s between the two expert annotations. A seizure hour was confirmed if there was at least one confirmed electrographic seizure within that hour.

For all neonates, an hourly seizure record form was prospectively completed at the cot side by the clinical team at each hospital during real-time monitoring. If any seizures (clinical, electrographic, or both) were recognised in a single hour by the local clinical team, the form was annotated accordingly. If no seizures were noted in that hour, the form was marked as “no seizures recorded”. A neonate was considered identified by the clinical team as having seizures (neonate with seizure) if at least 1 h was marked on the seizure record form or at least one therapeutic dose of an antiseizure medication was given during the investigation. A seizure hour was considered to be identified by the clinical team if the seizure record form was marked or antiseizure medication was given in that hour or the hour after. A seizure hour was considered to be a false detection if the form was marked in that hour and the experts did not annotate any seizures in that hour or the hour before. For the clinical identification of seizures, we used both the antiseizure medications given during that hour or the hour immediately after an electrographic seizure and the annotations on the seizure record form, which accounted for any missing documentation on the form.

### Outcomes

For the detection of neonates with seizures, the primary outcomes were sensitivity (percentage of seizure neonates correctly identified by the clinical team), specificity (percentage of non-seizure neonates correctly identified by the clinical team), and false detection rate (percentage of neonates classified as seizure neonates by the clinical team who did not have electrographic seizures). For the detection of seizure hours, the primary outcomes were sensitivity (percentage of seizure hours correctly identified by the clinical team) and false detection rate (percentage of hours classified as seizure hours by the clinical team that were not seizure hours). The secondary outcomes were summary measures of seizure burden (total seizure burden, maximum hourly seizure burden, and median seizure duration) and number of inappropriate antiseizure medications given. Administration of an antiseizure medication was considered to be inappropriate if an antiseizure medication was given with no confirmed electrographic seizure in that hour or the hour before administration (which allows for time to prepare and administer the antiseizure medication). The post-hoc outcomes were sensitivity (percentage of seizure hours correctly identified by the clinical team) based on day of the week (weekdays, from Monday to Friday, and weekends, Saturday and Sunday) and time of the day (day shift, from 0800 h to 2000 h, and night shift, from 2000 h to 0800 h).

### Statistical analysis

To demonstrate superiority of the intervention group in terms of true detections, a sample size of 33 neonates with seizures per group was necessary to detect an absolute difference of 25% in mean sensitivity between groups, assuming an SD of 35%, a power of 80%, a level of significance of 5%, and a two-tailed test. Assuming that 40% of the neonates who were monitored would have confirmed electrographic seizures, we estimated that 83 neonates per group (total n=166) would be required. The seizure status of each neonate was only confirmed after the evaluation of their EEG by a neurophysiologist. After 50% of the planned sample size was recruited, their EEGs were annotated and 25% had confirmed electrographic seizures. On the basis of this finding, the sample size requirement was increased to 132 neonates per group (total n=264) to account for the difference in estimated and actual proportion of neonates with seizures.

We describe continuous variables using median (IQR) and categorical variables using frequency (%). For each group (algorithm and non-algorithm), we calculated estimates of the primary and secondary outcomes and their corresponding 95% CIs. We also calculated differences between the groups (95% CIs) for each outcome. To account for stratified randomisation by hospital and the within-infant clustering of infant hours (for the detection of seizure hours), we calculated bias-corrected bootstrap 95% CIs (based on 100 000 iterations). For bootstrapping, neonates were divided into 16 clusters on the basis of their group allocation and the hospital to which they were admitted. For each iteration, we generated a bootstrap sample of neonates from each cluster (using simple random sampling with replacement), combined the bootstrap samples, and calculated the outcome for each group (algorithm and non-algorithm) and the difference in outcomes between the two groups. We calculated point estimates from the original data. We did post-hoc comparisons of detection of seizure hours between the two groups based on day of the week (weekdays, from Monday to Friday, and weekend, Saturday and Sunday) and time of the day (day shift, from 0800 h to 2000 h, and night shift, from 2000 h to 0800 h) using logistic regression models with an interaction term. For each outcome, neonates were analysed according to their randomisation group and neonates were excluded if they had missing data on that outcome. We did statistical analyses using Stata (version 15.0).

This study is registered with ClinicalTrials.gov, NCT02431780.

### Role of the funding source

The funders of the study had no role in study design, data collection, data analysis, data interpretation, or the writing of the report. The corresponding author had full access to all the data and had final responsibility for the decision to submit for publication.

## Results

**Figure 1:**
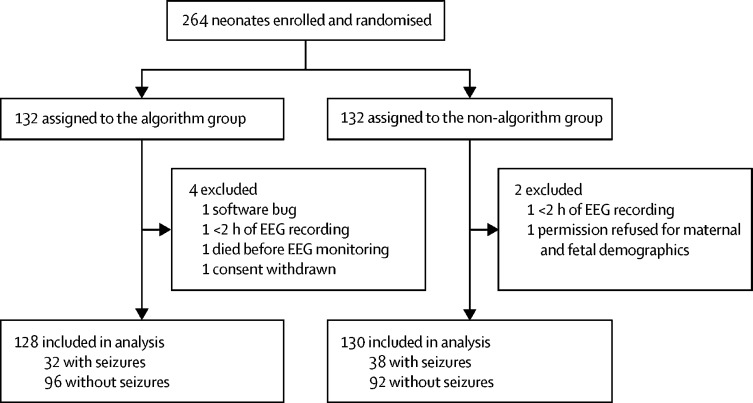
Trial profile EEG=electroencephalography.

Between Feb 13, 2015, and Feb 7, 2017, 132 neonates were randomly assigned to the algorithm group and 132 to the non-algorithm group. Six neonates (four from the algorithm group and two from the non-algorithm group) were excluded from the analysis (figure 1). Hence, 258 neonates (128 in the algorithm group and 130 in the non-algorithm group) were included in the study analysis. Neonates in both groups were similar in terms of clinical characteristics and EEG monitoring (table 1).

**Table 1:** Characteristics of neonates included in the analysis

		**Algorithm group (n=128)**	**Non-algorithm group (n=130)**
**Clinical characteristics**			
Corrected gestational age, weeks		40 (39–41)	40 (39–41)
Birthweight, g		3465 (3130–3813)	3350 (2958–3800)
Sex			
	Male	76 (59·4%)	79 (60·8%)
	Female	52 (40·6%)	51 (39·2%)
Apgar score at 5 min*		6 (4–9)	6 (4–9)
Therapeutic hypothermia			
	Cooled	69 (53·9%)	59 (45·4%)
	Uncooled	59 (46·1%)	71 (54·6%)
Final diagnosis			
	Mild hypoxic ischaemic encephalopathy	18 (14·1%)	14 (10·8%)
	Moderate hypoxic ischaemic encephalopathy	35 (27·3%)	31 (23·8%)
	Severe hypoxic ischaemic encephalopathy	21 (16·4%)	11 (8·5%)
	Stroke	15† (11·7%)	17‡ (13·1%)
	Metabolic or genetic disorder	10§ (7·8%)	13¶ (10·0%)
	Suspected seizures (unconfirmed)	6 (4·7%)	14 (10·8%)
	Perinatal asphyxia without clinical encephalopathy	4 (3·1%)	11 (8·5%)
	Sepsis or meningitis	6‖ (4·7%)	8 (6·2%)
	Intracranial haemorrhage	3 (2·3%)	2 (1·5%)
	Other	10** (7·8%)	9†† (6·9%)
**EEG monitoring during study**			
Age at start of study‡‡, h		32·1 (13·6–61·3)	28·0 (14·0–68·8)
Duration of cEEG monitoring, h		48·6 (26·1–83·7)	54·9 (22·3–86·1)
Total duration of cEEG monitoring, h		6746·7	7080·8

Data are median (IQR) or n (%) unless otherwise stated. cEEG=continuous conventional electroencephalography.

* 119 infants in the algorithm group and 123 infants in the non-algorithm group had data (data were missing in clinical notes for 16 infants).

† Three infants also had mild hypoxic ischaemic encephalopathy, two also had moderate hypoxic ischaemic encephalopathy, and one also had severe hypoxic ischaemic encephalopathy.

‡ One infant also had mild hypoxic ischaemic encephalopathy, two also had moderate hypoxic ischaemic encephalopathy, and one also had severe hypoxic ischaemic encephalopathy.

§ One infant also had severe hypoxic ischaemic encephalopathy.

¶ One infant also had mild hypoxic ischaemic encephalopathy and one also had severe hypoxic ischaemic encephalopathy.

‖ One infant also had mild hypoxic ischaemic encephalopathy.

** Six infants had transient metabolic deficit, two had brain malformation, one had multiple congenital abnormalities, and one had persistent pulmonary hypertension of the newborn.

†† Five infants had seizures of unknown origin, two had congenital cardiac anomaly, one had transient metabolic deficit, and one had brain malformation.

‡‡ Start of study is defined as time of randomisation or time EEG monitoring commenced (whichever was later).

The percentage of neonates with electrographic seizures was similar in both groups (32 [25·0%] of 128 in the algorithm group and 38 [29·2%] of 130 in the non-algorithm group; table 2). The primary outcome of measures of diagnostic accuracy (sensitivity, specificity, and false detection rate) for recognition of a neonate with seizures were not significantly different between the two groups (table 2). Sensitivity was 81·3% (95% CI 66·7–93·3) in the algorithm group and 89·5% (78·4–97·5) in the non-algorithm group; specificity was 84·4% (95% CI 76·9–91·0) in the algorithm group and 89·1% (82·5–94·7) in the non-algorithm group; and the false detection rate was 36·6% (95% CI 22·7–52·1) in the algorithm group and 22·7% (11·6–35·9) in the non-algorithm group. In the algorithm group, all six neonates with seizures who were not identified by the clinical team had a total seizure burden of 40 min or less. In the non-algorithm group, three of the four neonates with seizure not identified had a total seizure burden of 40 min or less.

**Table 2:** Comparison of diagnostic accuracy of algorithm and non-algorithm groups for detection of seizure neonate

	**Algorithm group (n=128)**	**Non-algorithm group (n=130)**	Difference (95% CI*)
Number of neonates with seizures	32	38	..
Number of true positives†	26	34	..
Number of true negatives‡	81	82	..
Number of false positives§	15	10	..
Number of false negatives¶	6	4	..
Sensitivity‖ (95% CI*)	81·3% (66·7 to 93·3)	89·5% (78·4 to 97·5)	−8·2% (−25·0 to 7·7)
Specificity** (95% CI*)	84·4% (76·9 to 91·0)	89·1% (82·5 to 94·7)	−4·8% (−14·1 to 4·6)
False detection rate†† (95% CI*)	36·6% (22·7 to 52·1)	22·7% (11·6 to 35·9)	13·9% (−5·2 to 32·7)

* Bias-corrected.

† Seizure neonate correctly classified as a seizure neonate by the clinical team.

‡ Non-seizure neonate correctly classified as a non-seizure neonate by the clinical team.

§ Non-seizure neonate incorrectly classified as a seizure neonate by the clinical team.

¶ Seizure neonate incorrectly classified as a non-seizure neonate by the clinical team.

‖ Percentage of seizure neonates correctly classified (ie, true positives among total true positives and false negatives).

** Percentage of non-seizure neonates correctly classified (ie, true negatives among total true negatives and false positives).

†† Percentage of neonates classified as seizure neonates by the clinical team who did not have seizures (ie, false positives among total true and false positives).

**Figure 2:**
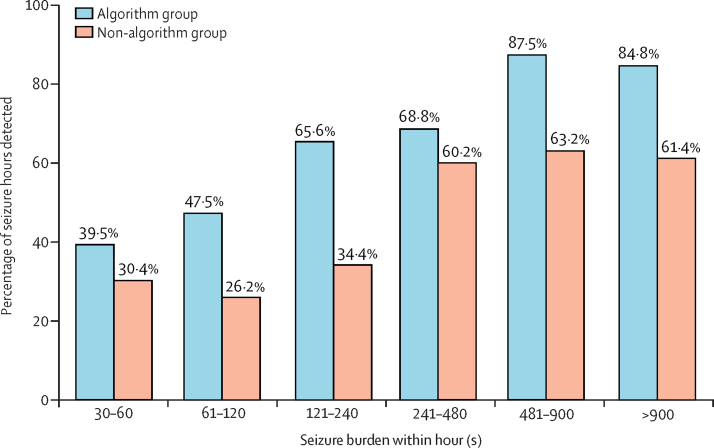
Percentage of seizure hours detected, by total seizure burden within the hour

Overall, there were 659 h in which confirmed EEG seizures occurred (268 h in the algorithm group and 391 h in the non-algorithm group). The percentage of seizure hours identified was significantly higher in the algorithm group (177 [66·0%; 95% CI 53·8–77·3] of 268 h in the algorithm group *vs* 177 [45·3%; 34·5–58·3] of 391 h in the non-algorithm group; difference 20·8% [3·6–37·1]; table 3). For both groups, identification of seizure hours increased with increasing total seizure burden within the hour (figure 2).

**Table 3:** Comparison of detection of seizure hours between algorithm and non-algorithm groups

		**Algorithm group**				**Non-algorithm group**				Difference in sensitivities (95% CI*)
		Number of neonates with seizures	Number of seizure hours	Number of seizure hours identified	Sensitivity† (95% CI*)	Number of neonates with seizures	Number of seizure hours	Number of seizure hours identified	Sensitivity†	(95% CI*)
Overall		32	268	177	66·0% (53·8–77·3)	38	391	177	45·3% (34·5–58·3)	20·8% (3·6–37·1)
Day of the week										
	Weekdays (Monday–Friday)	27	197	125	63·5% (52·4–74·1)	35	331	155	46·8% (35·9–59·6)	16·6% (0·1–32·3)
	Weekend (Saturday–Sunday)	13	71	52	73·2% (44·7–87·3)	10	60	22	36·7% (20·7–54·2)	36·6% (4·4–64·3)
Time of the day										
	Day shift (0800 h–2000 h)	25	128	91	71·1% (53·8–85·5)	31	191	92	48·2% (38·6–59·6)	22·9% (3·2–41·2)
	Night shift (2000 h–0800 h)	27	140	86	61·4% (49·6–72·7)	31	200	85	42·5% (30·3–56·3)	18·9% (1·1–35·9)

* Bias-corrected.

† Percentage of seizure hours correctly classified as seizure hours by the clinical team.

**Figure 3:**
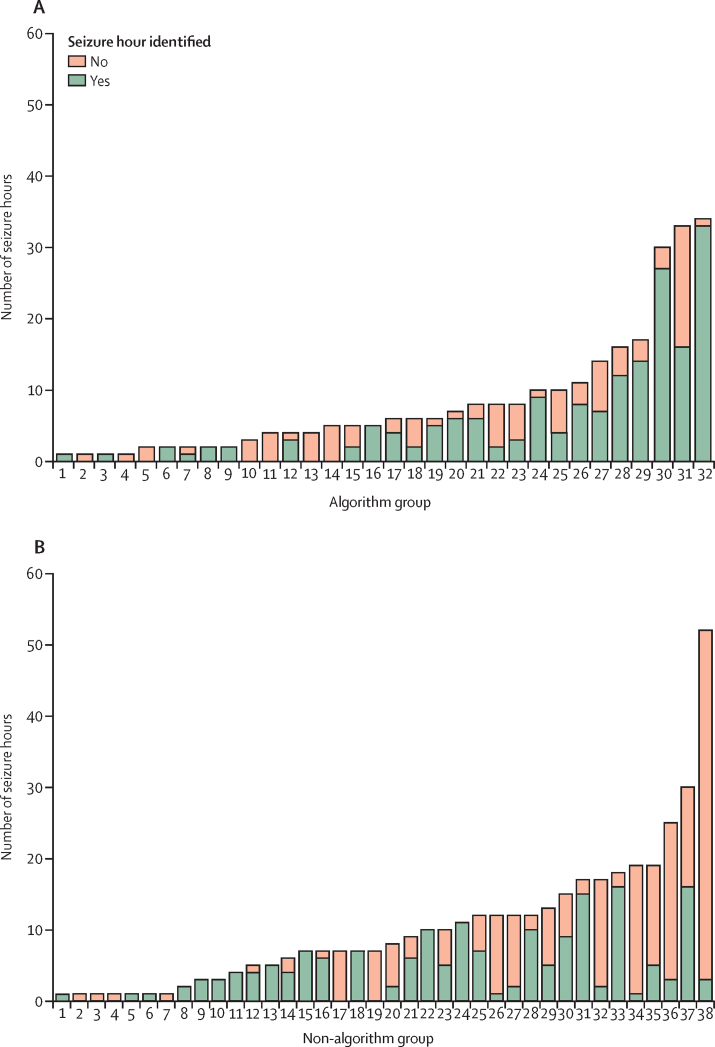
Number of seizure hours in neonates who had seizures in the algorithm group (A) and non-algorithm group (B) Each bar represents a neonate.

Seven neonates from the algorithm group and six neonates from the non-algorithm group had no seizure hour correctly identified (figure 3). 177 (38·4%) of 461 of the seizure hours detected on the seizure record form were not marked as seizure hours by the EEG experts, indicating that, although seizures were suspected by the clinical team, no electrographic evidence of seizures was observed (false detection). The false detection rate on the seizure record form did not differ between the groups (97 [39·3%] of 247 h in the algorithm group *vs* 80 [37·4%] of 214 h in the non-algorithm group; difference 1·9% [95% CI −14·0 to 18·6]). No significant differences were found between the groups regarding the secondary outcomes of seizure characteristics (total seizure burden, maximum hourly seizure burden, and median seizure duration) and percentage of neonates with seizures given at least one inappropriate antiseizure medication (37·5% [95% CI 25·0 to 56·3] *vs* 31·6% [21·1 to 47·4]; difference 5·9% [–14·0 to 26·3]; table 4).

**Table 4:** Secondary outcomes

		**Algorithm group**	**Non-algorithm group**	Difference (95% CI*)
**Neonates with seizures**				
Number of neonates with seizures		32	38	..
Seizure characteristics				
	Total seizure burden, min	32·3 (17·0 to 45·1)	46·8 (20·6 to 63·5)	−14·5 (−37·8 to 15·9)
	Maximum seizure burden, min/h	13·6 (11·0 to 23·5)	12·9 (9·1 to 20·6)	0·7 (−7·4 to 9·8)
	Median seizure duration, s	102 (91 to 182)	108 (77 to 184)	−6 (−84 to 54)
At least one inappropriate antiseizure medication†		12	12	..
	Percentage (95% CI)	37·5% (25·0 to 56·3)	31·6% (21·1 to 47·4)	5·9% (−14·0 to 26·3)
**Neonates without seizures**				
Number of neonates without seizures		96	92	..
At least one inappropriate antiseizure medication‡		10	4	..
	Percentage (95% CI)	10·4% (6·3 to 17·7)	4·3% (2·2 to 9·8)	6·1% (−0·3 to 13·5)

Data are median (bias-corrected 95% CI) unless otherwise stated.

* Bias-corrected.

† In the algorithm group, nine infants were given one inappropriate antiseizure medication and three were given two inappropriate antiseizure medications; in the non-algorithm group eight infants were given one inappropriate antiseizure medication, three were given two inappropriate antiseizure medications, one was given three inappropriate antiseizure medications.

‡ In the algorithm group, nine infants were given one inappropriate antiseizure medication and one was given two inappropriate antiseizure medications; in the non-algorithm group, four infants were given one inappropriate antiseizure medication.

In the post-hoc comparison of detection of seizure hours between the two groups based on day of the week (weekday *vs* weekend), the interaction term was significant (p=0·038). Differences in the recognition of seizure hours between the algorithm group and non-algorithm group was greater at weekends than weekdays (table 3). For the time of the day comparison, the interaction term was not significant (p=0·535), indicating that differences in the recognition of seizure hours between the two groups did not depend on the time of the day.

## Discussion

To the best of our knowledge, this multicentre, randomised investigation is the first to clinically evaluate a neonatal seizure detection algorithm in real time at the cot side in the NICU. We used a large dataset (13 827 h of cEEG) in a term neonatal population, including a proportion of neonates with seizures of various causes. Although the algorithm did not enhance the identification of individual neonates with seizures, the recognition of seizure hours increased significantly when the ANSeR algorithm was used as a support for seizure identification (66·0% in the algorithm group *vs* 45·3% in the non-algorithm group), and this increase was greater at weekends than during weekdays.

Over the past 20 years, several research teams have developed and validated seizure detection algorithms for the neonatal population,^21^, ^23^, ^25^, ^26^, ^31^, ^34^ and these studies have been discussed in a review.^35^ ANSeR has a variable sensitivity threshold that can be set before use, given the trade-off between seizure detection and false alarms. In a previous study,^31^ we determined that a threshold range between 0·5 and 0·3 was suitable for clinical use, giving a seizure detection range of 52·6–75·0% and false alarm rate range of 0·04–0·36 false alarms per h. In this study, we chose a fixed threshold of 0·5 to prioritise a very low false alarm rate on the basis that more frequent false detections might degrade confidence in the algorithm and increase the likelihood that the alarm was silenced for further detection, negating its primary function to alert staff at the time of a potential seizure.

Lawrence and colleagues^22^ used the Recognize algorithm in a prospective, randomised pilot study to assess the feasibility and clinical impact of continuous aEEG in a NICU environment. Neonates from a single centre were randomly assigned to a blinded group in which aEEG and the algorithm were not visible to the clinical team (20 neonates) or a visible group in which clinicians could interpret the aEEG recording supported by the algorithm (20 neonates). Of the 25 neonates with seizures, 12 were recognised by the clinical team (seven in the visible group and five in the blinded group). The real-time seizure detection rate of the algorithm was 55% (615 of 1116 seizures), with an increase to 73% for seizures longer than 30 s and a false alarm rate of 0·09 false alarms per h. 34 neonates had conventional EEG together with aEEG detecting 426 seizures (in ten neonates), of which 323 (76%) seizures were detected by the aEEG and 103 (24%) seizures were missed due to the limited EEG montage. Although the study^22^ used a seizure detection algorithm in a live setting, it did not constitute a clinical trial of the algorithm (and this was not the intention), as output of both the algorithm and EEG were unavailable to the blinded group. The other commercially available seizure detection algorithm, the Gotman algorithm, was tested on post-acquisition EEG recordings and not in real time in a clinical setting.^21^

The current multicentre, randomised investigation was powered to detect 25% superiority of the ANSeR algorithm in terms of true seizure detection, including neonates at risk of seizures from all causes. All of our recruiting hospitals routinely use aEEG, and some hospitals were also familiar with conventional EEG. Regardless of previous experience, all clinical teams received training for the interpretation of the aEEG and cEEG, as well as the ANSeR algorithm. By using multichannel EEG for our algorithm, we increased seizure detection compared with previous reports of limited two-channel EEG monitoring.^22^ The current study showed no difference between the groups in diagnosis of a neonate with seizures, but this finding might be explained by the fact that all recruiting hospitals were experienced in EEG monitoring and interpretation. Ten neonates with seizures were not identified by the clinical team: six in the algorithm group and four in the non-algorithm group. Of the six neonates in the algorithm group, the algorithm did not alarm for two. Both neonates were diagnosed with moderate hypoxic ischaemic encephalopathy, with a supressed EEG background and low amplitude, localised central seizures, and a low total seizure burden (<40 min). The other four neonates were probably missed as a result of short seizure durations.

The percentage of seizure hours recognised by the clinical team was higher in the algorithm group than in the non-algorithm group. Although the absolute difference in sensitivities between groups (20·8% [95% CI 3·6–37·1]) was below the set threshold of 25%, this finding is important considering the association between seizure burden and adverse long-term outcomes.^12^, ^13^, ^14^, ^15^, ^16^ In addition to the ten neonates with seizures not identified, three others had no seizure hours correctly identified by the clinical team: one in the algorithm group (with seizures detected by the algorithm) and two in the non-algorithm group.

Consistent with the report previously discussed by Lawrence and colleagues,^22^ the seizure hour detection rate by the clinical staff increased with an increase in the seizure burden for both groups but remained superior in the algorithm group. Differences in hospital staffing between day and night shifts and between weekdays and weekends are documented in the literature;^36^, ^37^ therefore, we wished to examine the effect of time of day and day of the week on the performance of the algorithm in a post-hoc analysis. Although no difference was noted between day and night shifts, a significant difference was observed between weekdays and weekends, with seizures being less likely to be recognised during weekends without the support of the algorithm. This finding might be more reflective of the situation in NICUs with less experience in EEG interpretation and in which no EEG expertise exists readily. We found no significant difference between groups in terms of seizure burden and inappropriate use of antiseizure medication, indicating that the algorithm did not result in infants receiving unnecessary antiseizure medication, supporting our conservative sensitivity cutoff to limit false alarms.

Our investigation has some limitations. We analysed all seizures that had an overlap of 30 s between the two experts, which were used as the gold standard for seizure diagnosis. In doing so, we excluded seizures with a duration of less than 30 s from both groups, for which agreement between experts is poor.^33^ The analysis was done using seizure hour instead of looking at each individual seizure. This decision was pragmatic, as it would not have been feasible to ask hospital staff to record every single suspected seizure lasting 10 s or more. To ensure that the clinical investigation plan was acceptable to all NICU personnel, the clinical team was asked to mark the hour, rather than mark specific times for onset and duration of seizures. Because NICU environments can be very busy, we are aware that missing documentation does not necessarily mean that seizures were not recognised. To account for this, we also considered that seizures had been recognised if an antiseizure medication was given during that hour or the hour immediately after an electrographic seizure.

Attempts have been made in a growing number of clinical conditions to use large datasets to aid categorisation of patient phenotypes and to assign outcome risk or diagnosis.^38^, ^39^ This current investigation is one of the first to move beyond a proof-of-concept into a real-time clinical investigation. Many years of signal processing analysis, using large datasets of neonatal EEG, allowed us to develop a reliable neonatal seizure algorithm.^29^, ^30^, ^31^, ^40^ Newly developed machine-learning techniques have allowed us to make rapid developments in the reliability of the algorithm and to bring this technology to the cot side.^35^, ^40^ We have shown that machine-learning techniques can be successfully and safely implemented into the clinical care of vulnerable neonates. We hope that this progress will encourage researchers in other fields of neonatal care to consider these techniques to solve real clinical problems.

In conclusion, this clinical investigation was the first to assess the performance of a machine-learning algorithm for neonatal seizure detection in real time and in the real-world setting of busy NICUs throughout Europe. Although all participating hospitals were experienced in neonatal EEG and the clinical teams were generally comfortable in interpreting the aEEG or cEEG, the support provided by the ANSeR algorithm still had a considerable effect on the seizure recognition rate. Our experience suggests that the benefit provided by the ANSeR algorithm might be greater if it was made available to centres with less experience of interpreting neonatal EEG at the cot side, but further research is required. The use of cEEG monitoring has increased considerably as therapeutic hypothermia has become standard practice, which has driven the need for accurate and timely interpretation. Many guidelines that seek to identify babies who might be suitable for therapeutic hypothermia recommend using EEG criteria including seizures, making accurate interpretation imperative. Future work on the probability setting of the algorithm and personalising it for each baby will probably improve performance.

## Data sharing

It is currently not possible to share the ANSeR dataset. The data is a clinical dataset collected under a written proxy consent from the parents of participants in the ANSeR Clinical Investigation and permission for sharing or open data was not included in the consent form. Therefore, we do not have the agreement of the data owner to share the pseudo-anonymised data and under Irish Health Research Regulations an approval by the Health Regulation Consent Declaration Committee or re-consent would be required. Full anonymisation of the dataset is currently not possible because follow-up assessments are ongoing.
